# Supplementary material for: Inhibitory Potential of Bifidobacterium longum FB1-1 Cell-Free Supernatant against Carbapenem-Resistant Klebsiella pneumoniae Drug Resistance Spread
Source: Microorganisms. 2024 Jun 14;12(6):1203. doi: 10.3390/microorganisms12061203 (PMC11205332; doi:10.3390/microorganisms12061203)

Supplementary file

Oxford cup experimental phenomena

BAA–1705

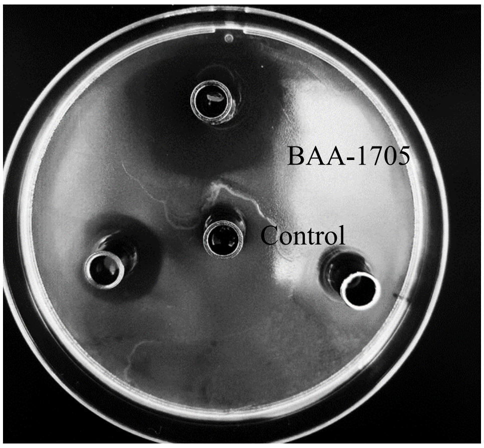

BNCC358281

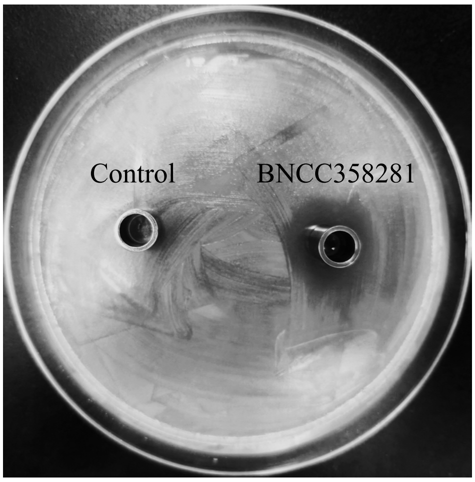

BNCC289979

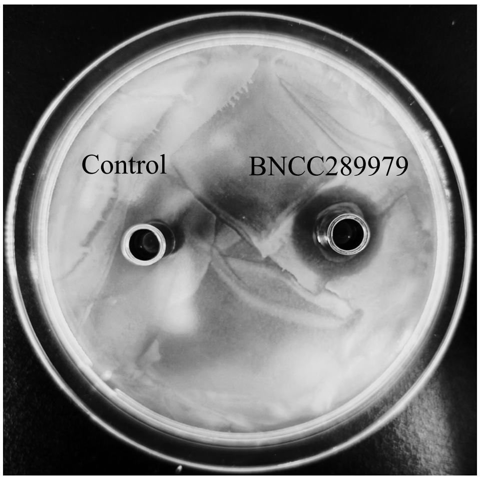

The MIC for BNCC358281, and BNCC289979

BNCC358281

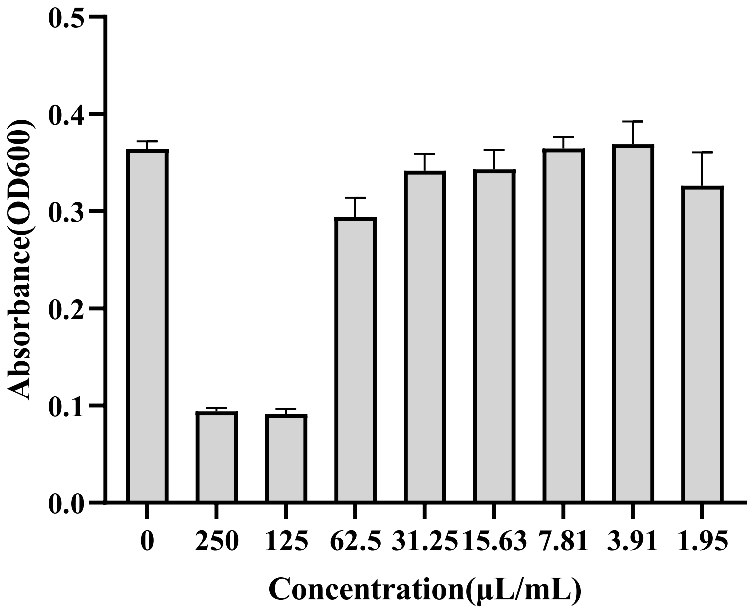

BNCC289979

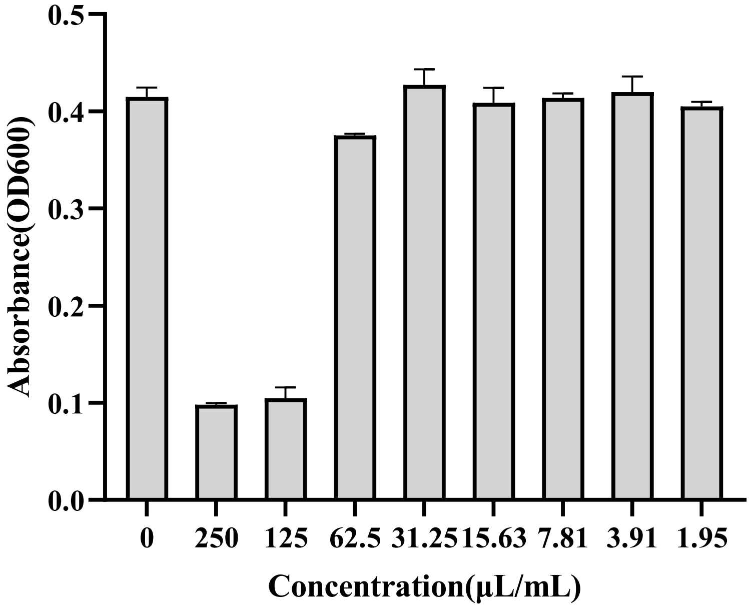

Supplement: Supplementary file 1 [file microorganisms-12-01203-s001.zip › microorganisms-2871007-supplementary.pdf]
